# Factors Influencing Community Engagement during Guinea Worm and Polio Eradication Endgames in Chad: Recommendations for “Last Mile” Programming

**DOI:** 10.4269/ajtmh.23-0635

**Published:** 2024-07-09

**Authors:** Maryann G. Delea, Lalique Browne, Severin Kaji, Adam J. Weiss, Ouakou Tchindebet

**Affiliations:** ^1^ The Carter Center, Guinea Worm Eradication Program, Atlanta, Georgia;; ^2^ The Carter Center, National Guinea Worm Eradication Program – Chad, N’Djamena, Chad;; ^3^ Ministère de la Sante Publique, Programme National d’Eradication de Ver du Guinée – Tchad, N’Djamena, Chad

## Abstract

Community engagement is a strategy commonly used in health and development programming. Many disease eradication programs engage with communities through different structures and mechanisms to detect, report, contain, and respond to the diseases they target. Qualitative operational research was conducted in a district of Chad co-endemic for both dracunculiasis (i.e., Guinea worm disease) and circulating vaccine-derived poliovirus to reveal factors influencing community engagement behavior in the context of eradication-related programming. Women and men from six communities and stakeholders from the local, district, and central levels were recruited to participate in focus group discussions and semi-structured in-depth interviews. A thematic analysis was performed to identify barriers and facilitators of community engagement. Barriers to community engagement included mistrust in exogenously established health program initiatives (i.e., initiatives designed by partners external to targeted program communities) resulting from negative past experiences with external entities and community groups and the lure of profit-motivating community engagement. Subgroup and intersectionality analyses revealed that gender and other identities influence whether and to what extent certain members of the community engage in a meaningful way. Facilitators of community engagement included leadership and the influence of authorities and leaders in community participation, perceived benefits of being engaged with community-based initiatives, and use of incentives to enhance community participation. Study findings may be used to inform the refinement of community engagement approaches in Chad and learning agendas for other “last mile” disease eradication programs.

## INTRODUCTION

Although it has no universally recognized definition in the context of global health, *community engagement* typically refers to collective efforts to promote the mutual exchange of ideas, information, and resources between members of a community and a health services provider or implementing organization (i.e., partners external to communities targeted by health programs).^1^ Community engagement involves but is not limited to the establishment of dynamic relationships and dialogue, shared involvement, and decision-making between community members and external partners.^2^ Enhancing community engagement is important not just so programs can help communities but also so community members can help programs by sharing knowledge of their communities and suggesting ways to solve problems from the communities’ unique perspectives. This programming approach gained traction with the Declaration of Alma-Ata in 1978, when the global health community declared the goal of “health for all” and emphasized the right and duty of the people to participate individually and collectively in the planning of their healthcare.^3^ For the past several decades, community engagement has been an integral part of health and development programming. It is viewed widely as a promising strategy to facilitate the uptake of program interventions and services to improve health.^2^

### Community engagement as a strategy for disease eradication.

Smallpox eradication programs created and supported robust surveillance systems that engaged both local health officials and communities in detecting, reporting, containing, and responding to smallpox cases in a timely manner.^4^ Building off the success of smallpox eradication, strategies that the public health community deemed effective within those national smallpox eradication programs, such as strong community engagement and community-based surveillance and response structures, were considered in the design of polio and Guinea Worm Eradication Program (GWEP) interventions.^5^

Although many strategies contribute to the gains forged by disease eradication efforts, the benefits of engaging communities in the planning and implementation of eradication-related interventions have been well documented.^6^ Community engagement is a fundamental component of disease eradication programming, particularly during eradication endgames when the last remaining cases of the diseases slated for eradication become more and more difficult to detect and contain. Consequently, there has been a recent call to action from the disease eradication sector to enhance community engagement and community ownership of disease eradication initiatives, with support from civil society organizations, to address “last mile” eradication endgame challenges.^7^

Poor community engagement in the context of eradication programs has revealed how a lack of appropriate community involvement can lead to misconceptions and noncompliance with the program interventions.^8^ For example, during 2003, vaccine hesitancy and the rejection of polio vaccines were observed in Nigeria owing to a lack of community engagement.^8^ Five northern Nigerian states boycotted the oral polio vaccine, claimed it to be unsafe, and contributed to the spread of false rumors about the vaccine.^9^ These setbacks to polio eradication efforts were later resolved by engaging community members in the acceptance of the vaccine.^9^

### Study rationale.

Both Guinea worm disease (GWD) and polio are slated for eradication.^10^ Although the Africa Regional Certification Commission recently certified the WHO African region free of wild polio,^11^ circulating vaccine-derived polioviruses (cVDPVs) have been detected in multiple countries, including Chad, where GWD remains endemic.^12^ Although available interventions may differ between Guinea worm (GW) and polio eradication programs (e.g., vaccination is an integral intervention for polio eradication programming, but interventions for the GWEP are largely behavioral in nature because there are no vaccines or therapeutic interventions for GWD), community engagement is an implementation strategy used by both. As such, various stakeholders working on these disease eradication initiatives have shared interests in increasing community ownership over surveillance, prevention, and response for both diseases in many of the same locations.

For the mutual benefit of both diseases, this qualitative operational research study was designed to elucidate the factors that influence whether and to what extent communities engage with GW and polio-related eradication initiatives in Chad (i.e., the Program National d’Eradication de Ver du Guinée-Tchad [PNEVG-T], Chad’s National GWEP, and the Expanded Program on Immunization [EPI]). The aim of this operational research was to generate evidence to identify gaps in community engagement strategies, yield data-driven insights, and inform recommendations for enhanced community engagement strategies within the context of these last mile eradication endgame initiatives.

## MATERIALS AND METHODS

### Study design.

During February–October 2021, the study team used qualitative research methods to examine factors influencing community engagement generally and within the context of GW and polio-related eradication initiatives in Chad. For the purposes of this investigation, we examined community engagement behavior broadly but also considered participation and involvement in community groups, institutions, and/or activities, voluntary or otherwise, as indicators of community engagement behavior.^13^

### Setting.

The Bongor District, located in southwestern Chad, was purposively selected for this operational research given it is an area co-endemic for cVDPV and GWD, and it is relatively heterogeneous on key dimensions related to coverage rates of polio vaccination and uptake of GW-preventive PNEVG-T interventions, history of and response to polio (cVDPV or otherwise) and GWD outbreaks, and history of community engagement. Within the Bongor District, the study team purposively selected six rural communities based on the same eligibility criteria used to select the targeted district; four were villages (Dor, Djarwaye, Ham, and Nahaina) and two were settlements where nomadic groups were located (Bariam and Kim). Discussions with key stakeholders at the district level in Bongor revealed a local context marked by low literacy rates, particularly in rural areas and among women, limited accessibility to health infrastructure for many communities, and strong preferences for traditional beliefs and practices. Data from local health centers accessed during the study indicated polio vaccination rates that were higher than national estimates (i.e., 100% of children in Ham, Nahaina, and Djarwaye and 88% of children in Dor had reportedly received the three required doses of oral polio vaccines, whereas an estimated 57% of children in Chad aged 1 year received the three required doses of polio vaccine in 2022).^14^

### Data collection.

The study team used two different qualitative data collection activities: focus group discussions (FGDs) and semi-structured in-depth interviews (IDIs). Prior to the execution of these qualitative data collection activities, the study team administered a full demographic survey with each community- and local-level participant that captured data on participant demographics and household characteristics (where applicable). Basic demographic data (e.g., gender, title, approximate age) were captured on each district and central-level participant. Relevant demographic data were collected from each participant to facilitate subgroup and intersectionality analyses. No participants were compensated financially for their engagement in the FGDs or IDIs.

#### Theoretically grounded inquiry: The use of behavioral theory and empirical evidence.

The study team leveraged the Capability, Opportunity, and Motivation Model for Behavior Change, an established behavioral framework^15^ and the underlying theories and empirical evidence on which the framework is grounded, to inform the design of the data collection tools (i.e., FGD and IDI guides) and analytical framework.^16^ Psychosocial constructs known to influence community engagement and health behaviors at individual, household, and community levels (e.g., trust, community commitment, past experiences)^17^^–^^19^ informed questions in data collection tools and deductive codes. Underlying domains and dimensions of empowerment, a documented factor of community engagement^2^ that may facilitate or attenuate community participation,^20^ were also considered, namely agency (time-use agency, decision-making, collective agency, leadership), institutional structures (relations, norms), and resources (assets).^21^^–^^23^

#### Focus group discussions.

The data collection team facilitated a series of FGDs among different segments of the population (i.e., subgroups) in the six communities targeted for data collection. The purpose of the FGDs was to generate data on community perspectives related to community engagement, mobilization, and involvement and participation in community-based surveillance and outbreak prevention and response activities (for both GW and polio); past experiences with community engagement initiatives; uptake of other eradication-related interventions; and aspects of program delivery.

Focus group discussion data collection teams consisted of one French/English–speaking consultant-facilitator (L. B. or S. K.) and one or two trained notetakers (i.e., facilitator-notetaker dyads/triads) who also translated the facilitator’s questions from French into the local Massa or Arabic dialect. Members of the data collection team had no interaction with study communities or participants prior to this operational research initiative. Women facilitator-notetaker dyads/triads conducted FGDs with women participants, and male facilitator-notetaker dyads/triads conducted FGDs with men participants. In addition to the field notes taken by the notetakers and facilitators, the team captured audio recordings of group discussions, with permission from participants. To protect participant anonymity and confidentiality, FGDs were conducted in secluded areas or private settings within the target village where no nonparticipants (aside from participants’ children) were present.

Over the course of three rounds of data collection, the team conducted a total of 15 FGDs on community engagement–related themes, each of which lasted approximately 1.5–2 hours and consisted of four to eight participants. Adults (i.e., individuals aged 18 years or older) who lived in villages or were residing in settlements targeted by the study with the following characteristics and who consented to participate were eligible for inclusion in the FGDs:

- Women who actively participated in community groups (*n* = 3 FGDs)
- Women who did not participate in community groups (*n* = 3 FGDs)
- Men who actively participated in community groups (*n* = 3 FGDs)
- Men who did not participate in community groups (*n* = 3 FGDs)
- Women or men who worked as community volunteers (i.e., community relais) for any organization based in the targeted villages or communities, including but not limited to PNEVG-T and/or EPI (*n* = 3 FGDs).

Individuals who were younger than 18 years of age, who were only visiting the villages or settlements targeted by the study, and/or who did not consent to participate in the study were ineligible for the FGDs according to these exclusion criteria.

Community relais were targeted as participants in the FGDs because both the PNEVG-T and the EPI recruited them as part of their community engagement strategies. The PNEVG-T community relais assist with the implementation of program activities, including but not limited to active surveillance for GWD, encouraging community members to report signs and symptoms of GWD, and counseling households on adopting GW-preventive behaviors. The PNEVG-T community relais received small compensation on a monthly basis for their engagement with the program. The EPI engages two types of community relais—health center community relais who are regularly involved in health center activities, including but not limited to vaccination, and community relais who are selected on occasion to support with mass vaccination campaigns. Health center community relais reside in villages within the health center’s catchment area, but they do not receive renumeration for their engagement. Community relais who are selected on occasion to support with mass vaccination campaigns can be selected from outside the community, and they receive small compensation for their engagement in the campaigns.

The data collection team used purposive and snowball sampling methods to identify and recruit participants for the FGDs. Snowball sampling is a sampling method that involves one individual being asked to provide the names of one or more potential participants, with the sample growing like a snowball as more potential participant referrals are provided per individual.^24^ The team sought heterogeneity in the selection of participants with regard to selecting participants who resided in different households and household concessions within each community, inhabited different neighborhoods within target communities, and lived varying distances from the health center. To ensure this diversity in FGD participants, the team developed community maps that displayed the different neighborhoods within the communities, with the assistance of health post staff, community leaders, and volunteers. The team then traveled to each neighborhood to recruit FGD participants. The study team carried out participant recruitment during 1–2 full days at the beginning of each round of data collection.

#### In-depth interviews with key stakeholders.

The data collection team also facilitated semi-structured IDIs with community members and key stakeholders from the local, district, and central levels who were identified during a stakeholder analysis that preceded the IDI data collection phase. The purpose of the IDIs was to examine different topical areas across the various target audiences, including those related to household- and community-level power dynamics; specific strengths and weaknesses of community engagement approaches; suggestions for improvements to existing strategies and approaches; and ideas for novel opportunities, strategies, and approaches to enhance community engagement.

The IDI data collection teams consisted of one French/English–speaking consultant-facilitator (L. B. or S. K.) and one trained notetaker (i.e., facilitator-notetaker dyads) who had no interaction with study communities or participants prior to this operational research initiative. In-depth interviews conducted at the district and national levels were conducted in French; when needed (e.g., with community members and some local-level stakeholders), the notetaker translated the facilitator’s questions from French into the local Massa or Arabic dialect. For the most part, women facilitator-notetaker dyads conducted IDIs with women participants, and male facilitator-notetaker dyads conducted IDIs with men participants. In addition to the field notes taken by the notetakers and facilitators, the team captured audio recordings of interviews, with permission from participants. To protect participant anonymity and confidentiality, interviews were conducted in secluded areas or private settings that were convenient to the participants (e.g., private offices, household compounds) where no nonparticipants were present.

The data collection team conducted a total of 32 IDIs, each of which lasted approximately 1–1.5 hours. Individuals meeting the following criteria who consented to participate were eligible for IDIs:

1. Target audiences for topics related to household- and community-level power dynamics that may influence decision-making around engagement with community health programs, namely participation and leadership in community groups/organizations (voluntary and nonvoluntary positions), movement, and interactions outside the house (i.e., freedom of movement and time-use agency):
  - Community members who were leaders (*n* = 6 IDIs; 3 men, 3 women)
  - Community members who were not leaders (*n* = 6 IDIs; 3 men, 3 women)
2. Target audiences for topics related to community engagement approaches (for polio/EPI and GW/PNEVG-T); suggestions for improvements to existing strategies and approaches; ideas for novel strategies and approaches:
  - Local-level stakeholders (*n* = 9 IDIs; 3 members of health committees [i.e., Health Committee – COSAN and Health Center Management Committee – COGES], 3 vaccination agents, 3 PNEVG-T agents)
  - District-level stakeholders (*n* = 5 IDIs) and
  - Central-level stakeholders (*n* = 6 IDIs)

Individuals who did not meet the aforementioned criteria, who participated in FGDs, and/or who did not consent to participate in the study were ineligible for the IDIs according to these exclusion criteria.

As with the FGDs, the team used purposive and snowball sampling methods to identify and recruit participants for IDIs. For the selection of community leaders, particularly men, the study team ensured that various segments of the community were reflected by way of recruitment of religious leaders as well as village chiefs and other community group leaders. In study communities, women only held leadership roles in community groups. Service delivery agents and surveillance officers working in the targeted district were identified and recruited for IDIs.

The data collection team deployed a participatory free listing exercise^25^ at the start of IDIs with women and men leaders and non-leaders, whereby the participants were asked to describe what community engagement meant to them and to list examples of community engagement activities. After participants provided their responses, the study team then explained that “community engagement for our context is when an organization or government agency comes into a community and engages community members in activities.” The data collection team then asked the participants to list their experiences with these types of community engagement activities. The study team designed the participatory free listing exercise to help reveal the cultural salience of community engagement and understand community perspectives regarding what constitutes community engagement.

#### Discussion and interview guide development and refinement, debriefings, and transcription and translation of audio files.

Details related to the development and refinement of discussion and interview guides are presented in the Supplemental Materials along with information related to debriefings and the transcription and translation of audio files.

## ANALYTICAL METHODS

### Thematic analyses.

**Figure 1.**
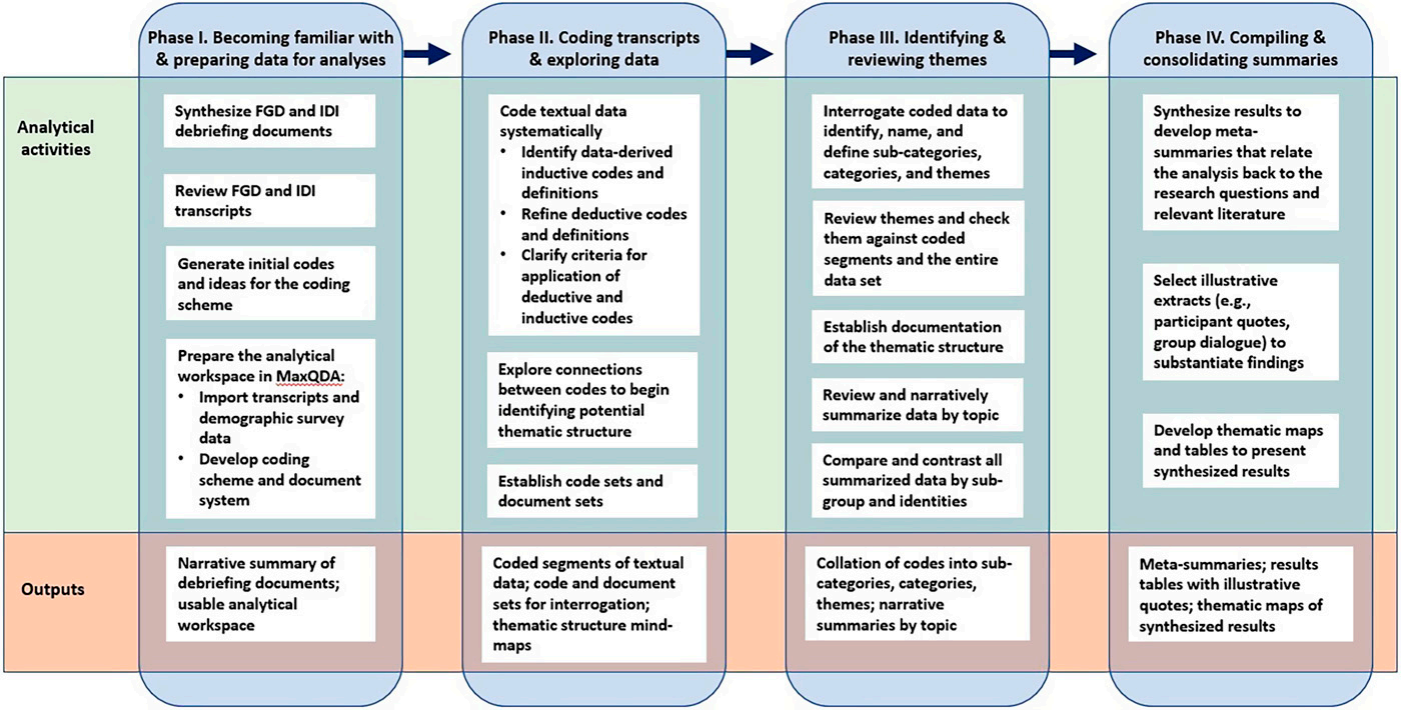
Summary of thematic analysis workflow. FDG = focus group discussion; IDI = in-depth interview.

The study team used a multiphased approach to conduct thematic analyses on the data from FGDs and IDIs.^26^ This approach is summarized in Figure 1 and detailed below.

The study team used the results of a synthesis of debriefing documents and an initial review of a subset of transcripts to refine the conceptualization and definitions of deductive codes that were informed by theory and prior evidence and to initiate the development of inductive codes derived from the data.^27^^,^^28^ Two team members (L. B. and S. K.) coded the textual data from FGD and IDI transcripts in MaxQDA (Berlin, Germany; VERBI Software, 2021) using a line-by-line coding process.^29^ Prior to the systematic coding of all transcripts, the two analysts independently coded a subset of three transcripts, after which coded segments were compared to assess intercoder reliability^30^; the assessment revealed high intercoder reliability. The analysts continued developing the codebook by incorporating additional inductive codes as they were identified during the coding process.

Once all transcripts were coded, the two analysts began interrogating the coded data, using mind-maps as a foundation to identify, name, and define subcategories, categories, and themes. To compare and contrast results by subgroup and identities, the two analysts used insight-driven population segmentation (i.e., subgroup) and intersectionality analyses.^31^ The analysts conducted subgroup analyses to examine behaviors, behavioral factors, and preferences that different types of people (e.g., women, men, transient populations, community leaders, individuals who participate in community groups/activities and those who do not, people living in different study sites) have when it comes to community engagement. The analysts examined the potential for differential influence of intersectionalities (e.g., interactions of gender, marital status, education, age, religion, other identities) on these behaviors as well.^31^ In the final phase of data analysis, the two analysts synthesized the thematic analysis results to develop meta-summaries, selected illustrative extracts (e.g., participant quotes, group dialogue) to substantiate findings, and developed a thematic map to visually present the synthesized results.

#### Summarizing community engagement behavior and conducting nonparticipant persona analyses.

The two analysts created meta-summaries to describe general community engagement behavior and then conducted a doer/non-doer analysis to elucidate the archetypical demographic profile, or behavioral persona,^32^ of an individual who did not practice community engagement behaviors (e.g., did not serve as a community volunteer for or otherwise engage with GW and polio-related initiatives). Understanding the persona, or archetypical demographic profile of nonengaged individuals adds value by providing program administrators with information they can use to further refine the targeting of interventions and the design of intervention approaches to reach individuals with similar profiles and improve their participation for more inclusive community engagement.

## RESULTS

A total of 130 participants engaged in the FGDs (*n* = 98) and IDIs (*n* = 32), 20 of whom were stakeholders from the central (*n* = 6), district (*n* = 5), and local (*n* = 9) levels, and 110 of whom were participants from the community level. Table 1 summarizes participation by study activity, and Supplemental Table 1 details participant characteristics and demographics.

**Table 1:** Participation by study activity

Participant Profile	Study Activity	Number of Participants
Women Active in Community Groups	3 FGDs	18 Participants Total
Women Not Active in Community Groups	3 FGDs	20 Participants Total
Men Active in Community Groups	3 FGDs	22 Participants Total
Men Not Active in Community Groups	3 FGDs	23 Participants Total
Community Volunteers (i.e., community relais)	3 FGDs	15 Participants Total
Central-Level Stakeholders	6 IDIs	6 Participants
District-Level Stakeholders	5 IDIs	5 Participants
Local-Level Members of Health Committee (COSAN), Health Center Management Committee (COGES)	3 IDIs	3 Participants
Local-Level PNEVG-T Agents	3 IDIs	3 Participants
Local-Level Vaccination Agents	3 IDIs	3 Participants
Women Community Leaders	3 IDIs	3 Participants
Women Community Members Who Are Not Leaders	3 IDIs	3 Participants
Men Community Leaders	3 IDIs	3 Participants
Men Community Members Who Are Not leaders	3 IDIs	3 Participants

COSAN = Health Committee; COGES = Health Center Management Committee; FDG = focus group discussion; IDI = in-depth interview; PNEVG-T = Program National d’Eradication de Ver du Guinée-Tchad.

### Conceptualizations of community engagement.

For the free listing exercise used during IDIs with women and men leaders and non-leaders, when interviewers first asked participants to describe what community engagement meant to them, many participants described that community engagement revolves around collectives and groups of people who form organically within the community. Salient responses referred to agricultural collectives and working as a group to produce more meaningful change in the community because working alone is not as efficient in terms of income generation and community development.

“We work together because alone, you can’t do much. If the work that the person does is beneficial and brings development, you can come in to make your contribution so that things go well, right?’’ *– Community-level IDI participant from Dor, community leader, man, 56 years of age*

During all FGDs and the subset of IDIs in which the free listing exercise was not used, the data collection team provided, at the outset of the study activity, the operational definition of community engagement: “community engagement is when an organization or the government comes into a community and engages community members in activities.” Overall, these FGD and IDI participants perceived community engagement as help received *from* external donors for the development of their communities. Few participants, especially community volunteers and community leaders, defined community engagement as “engaging benevolently in development activities” and “local communities joining external entities to help the community.”

### Community engagement behavior.

**Figure 2.**
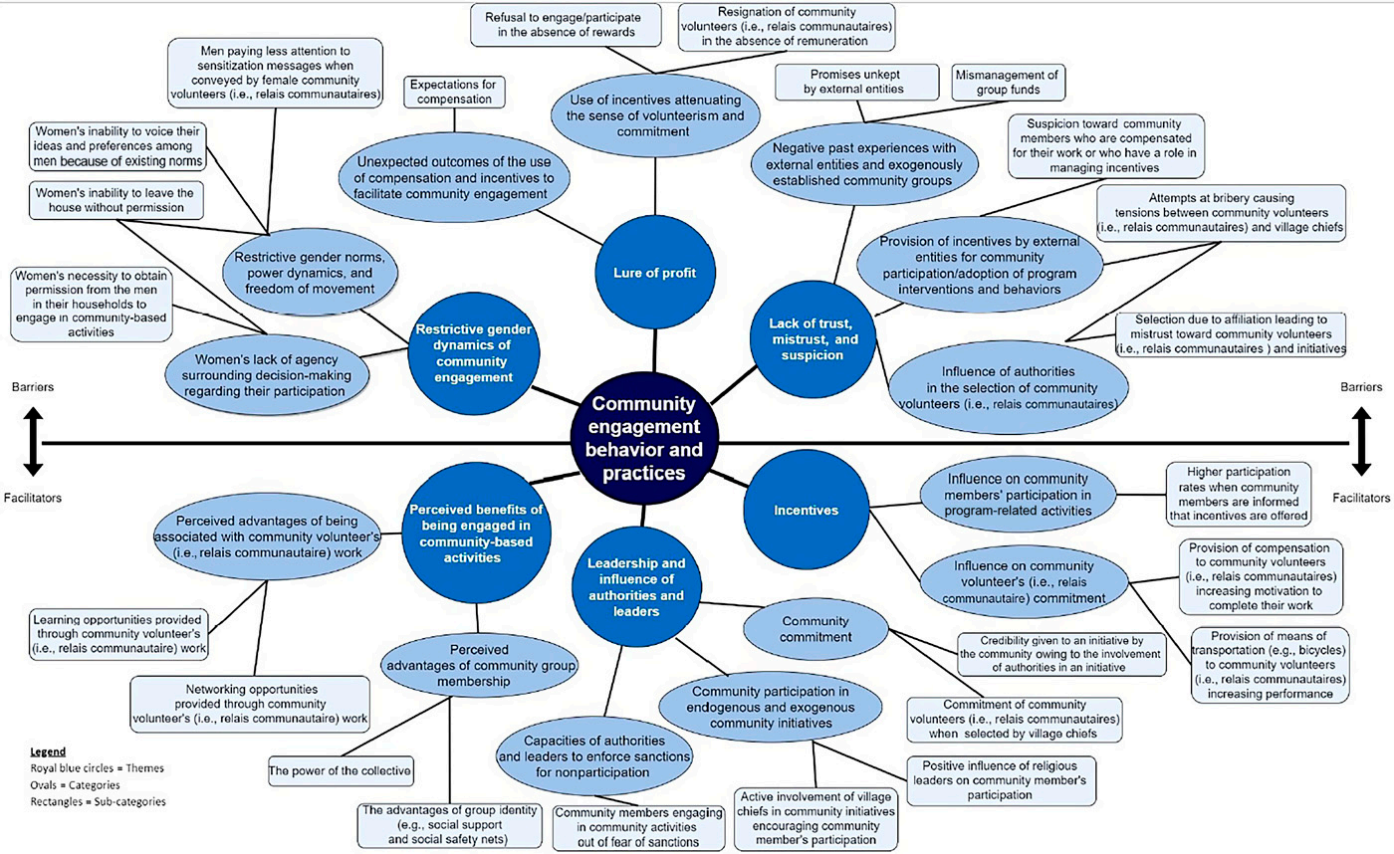
Thematic map of factors influencing community engagement behavior. Relais communautaires = community volunteers.

Within study communities, participation in community groups occurred informally and primarily through endogenously established collectives of people who come together to cooperate on farming activities. More formal community engagement constitutes participation in both endogenously and exogenously established community development associations, community involvement in health infrastructure (e.g., COSAN, COGES), and work as community volunteers (i.e., community relais) with different organizations or initiatives. Community engagement and participation were found to be influenced by different factors that serve as barriers to and facilitators of these behaviors. We summarize these factors in Figure 2 and provide further information and context in the narrative summaries that follow.

#### Persona of nonengaged individuals.

Doer/non-doer (i.e., participant/nonparticipant) persona analyses assessed the demographic profiles of 98 community-level participants from whom full demographic data were available: 53 of whom (54%) did participate in community groups and 45 of whom (46%) did not participate in community groups. Demographic data alone suggest that marital status did not heavily influence community group participation, but qualitative results from subgroup and intersectionality analyses revealed that gender, marital status, age, and educational attainment interacted with other psychosocial or structural factors (e.g., agency, access to resources) to limit the adoption of community engagement behaviors (e.g., participation in community groups, service as a community volunteer). As a result, the nonengaged/nonparticipant persona tended to reflect a younger married woman with little to no formal education who lacked agency (e.g., agency over her time-use, agency over her participation in community groups) and perhaps also lacked the freedom to move outside of the house independently.

### Factors influencing community engagement: Barriers.

#### Lack of trust, mistrust, suspicion.

Participants indicated that participation in community-based initiatives is influenced by community members’ past experiences related to collective engagement with external entities (e.g., organizations or individuals) and individual experiences with community groups. Negative past experiences created mistrust and suspicion among community members. Promises unkept by external entities and mismanagement of group funds were two salient sources of negative past experiences with external entities and exogenously established community groups.

“People come [into our communities] and say that they’ll help us, us who are in collectives. But they take all [the money] that we contribute and don’t come back to follow up. This attitude scares us, and we become suspicious.” *– Community-level FGD participant from Dor, member of a community group, woman, 37 years of age*

The provision of compensation and financial incentives by external entities has also contributed to tension and suspicion within communities. Participants indicated they were suspicious of community members who received or managed incentives (e.g., community volunteers, PNEVG-T agents, chiefs, heads of health centers) and the community volunteer selection process itself. Community members who are compensated for their work (e.g., community relais, PNEVG-T agents) or who have a role in managing incentives received by external entities (e.g., village chiefs, heads of health centers) are often suspected of mismanaging funds by other community members. Village chiefs reported that whenever there is a new project to be implemented in a community, chiefs are often suspected of having received gifts for the population but keeping everything for themselves and their relatives. The PNEVG-T agents reported that community members often have openly suspected them of keeping for themselves part of the rewards PNEVG-T provides community members for reporting GWD cases or infected animals. As a result of the suspicion, some community members refuse to allow PNEVG-T agents to access their compounds. Similarly, participants indicated that heads of health centers are often accused of deducting the compensation or financial incentives provided to vaccination agents.

Participants reported that involvement of authorities (e.g., village chiefs, head of health centers) in the selection of community volunteers (i.e., community relais) often results in selection due to affiliation (e.g., being known by or related to the village chief or head of the health center), which leads to the community mistrusting not only the selected community relais but also the initiative they are engaged to support.

“Instead of choosing two people per village as required, the chief chooses himself, chooses his wife or his son. […] These kinds of chiefs paralyze the work; the work cannot progress.” *– Central-level IDI participant, man, approximately 55 years of age*

Alleged attempts at bribery in which village chiefs require community volunteers to give them part of their compensation were reported as one reason why some chiefs choose their relatives to serve as community volunteers. Participants, particularly those from the central and district levels, also reported tension between chiefs and community volunteers who refused to share their financial incentives. To avoid the negative implications of selection by village chiefs, some participants suggested that community volunteers should be elected by the population and not appointed by the village chiefs.

#### Lure of profit.

Central-, district-, local-, and community-level participants reported that people partake in community initiatives primarily for a perceived profit. They also reported that community members refuse to engage or participate in the absence of gifts or rewards.

“[…] To summarize, I would say that it is mostly about motivation issue. People expect a financial motivation, even if they do not say it openly.” *– Central-level IDI participant, man, 55 years of age*

The employment of compensation and incentives as a strategy for enhancing community engagement has led to unexpected outcomes within communities, namely an expectation for compensation. Participants reported that there is an expectation within communities that people are compensated for community participation. These expectations reportedly result from the communities’ past and current experiences with different external organizations. Using gifts to enhance community involvement was reported as having culminated in an altered understanding of community engagement.

“When we go to a village, [people say] ‘when other[s] arrived here, they gave us this, they gave us that, and you just come to make sensitization; we don’t agree at all.” *– Local-level IDI participant, PNEVG-T agent, man, 45 years of age*

This barrier was particularly salient among community members and local-level stakeholders (e.g., community relais, community leaders, PNEVG-T agents, vaccination agents). These actors indicated that the negative influences of compensation and financial incentives have shaped the community’s attitudes toward participation in and engagement with various programs and initiatives.

Leaders and community members who were participants of community groups mentioned the absence of incentives as a reason why other community members did not participate in community-based activities. Non-leaders and community members who were not participants of community groups indicated the absence of financial incentives as a barrier to community participation. The employment of incentives has attenuated the sense of volunteerism and commitment within communities. Participants reported that community relais resign in the absence of renumeration, even when the volunteers indicated that their will to contribute to the development of their village was the reason they engaged as a community relais, which illustrates the lack of true volunteerism and commitment among community members to engage with various initiatives. In many instances, compensation or an incentive is a requirement for participation in community-based activities.

“Tell [the NGO which employs us as community relais] to give us money. Otherwise, we will give up as others did. We were many of us at the start [of the project], but we are currently just a few of us.” *– Community-level FGD participant from Dor, community volunteer, woman, 21 years of age*

It was also reported that the absence of financial incentives discourages volunteers and causes them to give up easily when faced with challenges in the field (e.g., rainfall, fatigue). Some community relais engaged in sensitization reported that community members often refuse to welcome them unless they offer gifts such as soap. There were also reports of households that refused to have children vaccinated because they had never been recruited as community relais to support vaccination activities and, as a result, had never benefited from related incentives.

*Restrictive gender dynamics of community engagement.* Gender dynamics of community engagement involve women’s lack of agency surrounding decision-making regarding their participation in community-based initiatives as well as gender norms, power dynamics, and control over women’s movements that restrict women’s engagement more broadly. The difference between men’s and women’s participation in community groups stems from the decision-making processes involved in joining such groups. Participants reported that, in general, women do not have independent decision-making authority and need to ask and obtain permission from the men in their households to join a community group, whereas men can decide for themselves whether they will join a group.

“If my wife wants to join a [community] group, I’m the one who authorizes it, and if I want to join a [community] group, I decide.” *– Community-level IDI participant from Djarwaye, non-leader, man, 50 years of age*

In general, men reported that women must obtain permission from men to join and participate in a community group, and few participants claimed that women have the freedom to join a group if they themselves choose to do so. Other participants emphasized that it is important for women to join a community group because groups are knowledge-sharing platforms and what women learn from their participation with community groups can be useful for the entire household.

Although participants reported that mixed-gender community groups exist, most groups are known to be gender specific. Participants also reported that women participated less in community mobilization activities than men. One reported reason for women participating less in community mobilization was because social mobilization activities bring together men and women, yet women cannot voice their ideas and preferences among men because of existing norms. However, it was reported that when community members are invited for community work for the maintenance of the health center, men participate less than women. Women community relais also mentioned that sensitization activities carried out by women are not effective with men because men pay less attention when a message is delivered by a woman. A district-level participant independently reported that women who try to conduct community sensitization activities as part of community engagement approaches face this challenge.

“A woman can go into a household [to sensitize]. The man may not accept. Because it is a woman who has come to talk. You see that!” *– District-level IDI participant, man, approximately 45 years of age*

There was a reported lack of agency over women’s freedom of movement (e.g., ability to leave the house) in general. Although both women and men described the limited agency of women, the data suggested that the power and influence of men over women varies. Women who reported that they had the freedom to participate in community groups and to leave the house when they wanted were participants in the “group leader” subgroup (i.e., these women were leaders of a community group). Married women and younger adult women reported having less agency over their own choices than widowed women and older women. This suggests that the power dynamics that have the strongest influence over women’s engagement and participation occur within the family sphere as opposed to the community sphere, though there likely are interactions between the two spheres, as some communities may be more conservative than others when it comes to women’s and girls’ empowerment.

### Factors influencing community engagement: Facilitators.

#### Leadership and influence of authorities and leaders.

Leadership and the influence of authorities and leaders over community participation in endogenous and exogenous community initiatives were indicated as facilitators of community engagement. These facilitators deal with community commitment and the capacities of authorities and leaders to enforce sanctions for nonparticipation (e.g., sanctions include the threat of being brought in front of authorities for not complying with vaccination recommendations and fines imposed by the village chief to community members who refuse to adopt disease-preventive practices).

Endogenous and exogeneous community initiatives were more successful when the village chief was actively involved in the design and/or follow-up of related activities. Religious leaders (e.g., pastors and imams) were also reported as having a positive influence on community members. Followers are likely to join an initiative when their leaders invite them or when they see their leaders actively participating in the initiative.

Involvement of leaders was found to increase commitment to a community-based activity.

“People follow what the chief says. If the chief is behind this eradication problem, I think the whole population is awake.” *– District-level IDI participant, man, approximately 45 years of age*

The positive influence of authorities was illustrated through the commitment of community relais selected by village chiefs and the credibility associated with the involvement of authorities in an initiative. The influence of the village chief reportedly motivates community volunteer work. When selected by the village chief, community volunteers will endeavor to remain committed and achieve satisfactory results to maintain the chief’s confidence. However, this point was less salient than the barrier related to the creation of mistrust that resulted from the village chief’s selection of community volunteers (i.e., community relais) according to affiliation rather than skill or competency.

Participants reported that the level of motivation and mobilization for an initiative are also influenced by the involvement of authorities and other leaders, including village chiefs. Inviting village chiefs and administrative authorities together with the population for social mobilization events related to an initiative is viewed as synonymous with credibility. When administrative and traditional authorities are involved in an initiative, community members perceive it as “the state’s initiative,” and such perceptions increase community participation and commitment. The involvement of village chiefs was reported as particularly important because of their proximity to the population. People usually respect decisions taken by their chiefs. Among authority figures, the influence of the village chief was reported as particularly prominent. Chiefs have the power to determine and enforce sanctions for nonparticipation in community-based initiatives. Participants reported that some people engage in community-based initiatives to avoid sanctions by the chief.

#### Incentives.

Incentives emerged as a salient facilitator of community engagement, as they reportedly influence community members’ participation in program-related activities as well as the motivation of community volunteers. Data from community-level participants suggest that incentives positively influence communities’ participation in community-based activities. Participants reported that participation rates were higher for social mobilization activities when community members were informed that gifts or incentives would be offered for their participation. The positive influence of incentives highlighted by community-level participants, including but not limited to community relais, tended to focus on the importance of incentives for successful community mobilization.

“Gifts quickly change people’s minds […]. If there’s a job to be done and there’s no money or gift, we call people to work and they won’t come. If there is a gift or money [offered], many will take part.” *– Community-level IDI participant from Djarwaye, woman leader, 40 years of age*

Data also suggest that financial incentives positively influence the performance and commitment of community volunteers. Community volunteers who are provided with a means of transportation (e.g., bicycles to use to facilitate the coordination of program activities) were reportedly more motivated to complete their work and were able to also use the bicycle for their own personal needs.

“In our case, for example, they’ve given us bikes to go around so much easier, and that really motivates us.” *– Community-level FGD participant from Djarwaye, community volunteer, man, 32 years of age*

Considering this connection between incentives and performance, many participants, particularly those from the central and district levels, suggested that community relais should be given financial incentives to stay motivated. The positive influence of incentives was mentioned by all participants, from the central to the community levels, but each subgroup of participants provided their different perspectives. Central- and district-level stakeholders and community volunteers themselves highlighted the importance of financial incentives provided to community volunteers to ensure they perform well.

#### Perceived benefits of being engaged in community-based activities.

Participants reported several perceived benefits of being engaged in community-based activities, which they reported as facilitators of community engagement. Perceived advantages of being associated with community volunteer work included the learning and networking opportunities the position provides. Perceived advantages of community group membership included the power of the collective and the advantages of group identity (e.g., social support and social safety nets).

Some participants who were community relais indicated that the position of community volunteer provides individuals with learning opportunities because the work allows them to acquire knowledge in a specific domain and learn skills that are often applicable to and beneficial for their private lives.

“[…] our work […] gives us lessons how to educate our children in the right way; it is good. But for someone who has not done it once, if his[/her] child suffers, he[/she] will not know what to do, he[/she] will stay quiet when the temperature rises […]. A person who has done it once would notice that the child’s breathing has changed and would automatically take him to the hospital, and if [the child] recovers, it is thanks to our [daily sensitization work…].” *– Community-level FGD participant from Dor, community volunteer, man, 20 years of age*

For community relais, incentives for participating in community-based activities are not necessarily financial. There are symbolic or immaterial incentives for engaging with the work as well. Participants reported that being a community volunteer often resulted in fame and prestige. For example, community volunteers are often called “docta” (meaning medical doctor) within the community.

Regarding participation in community groups specifically, participants indicated that an important factor that influenced their decision to participate was the perceived strength of a group. In general, participants reported joining community groups because they believed that it is more difficult for an individual to solve a problem than for a group of people. Participants reported the benefits attached to community group membership, including group identity and the social safety nets afforded by the group.

## DISCUSSION

This qualitative operational research study was designed to elucidate the factors that influence whether and to what extent communities engage with GW- and polio eradication–related initiatives specifically and health programs more broadly. Data generated through this operational research study suggest that an array of psychosocial, structural, financial, and contextual factors influence community engagement with disease eradication initiatives and health programs. Participants indicated barriers to community engagement included a lack of trust, mistrust, and suspicion of exogenously established health program initiatives resulting from negative past experiences with external entities and community groups and the lure of profit-motivating community engagement due to the use of incentives that attenuated the community’s innate sense of volunteerism and commitment. Subgroup and intersectionality analyses revealed that gender and other identities influence whether and to what extent certain members of the community are able to engage with community health programs and initiatives in a meaningful way. Facilitators of community engagement included the influence of authorities and leaders in community participation, the perceived benefits of being engaged with community-based initiatives, and the use of incentives to enhance community participation.

### Alignment of study findings with existing evidence.

Although some of the findings of this operational research uniquely contribute to the literature, many of our findings are consistent with existing evidence. As with other studies that have noted many different definitions of community engagement,^33^ we noted a divergence between community perspectives regarding the meaning of community engagement and the operational definition used for our study. We observed these differences in the definition of community engagement during the piloting phase. To account for these discrepancies, we incorporated the free listing exercise in the IDIs with women and men leaders and non-leaders to continue collecting data on community perspectives of the definition of community engagement while also explicitly stating our operational definition of community engagement during the other IDIs and FGDs.

Several other studies examining barriers and facilitators of community engagement within the context of polio-related initiatives and vaccination campaigns also found that mistrust, suspicion, and a lack of community commitment were barriers,^8^^,^^33^^–^^36^ whereas strong leadership was a facilitator.^34^ Our observations that household- and community-level power dynamics and restrictive gender norms influence women’s community engagement behavior in a negative way corroborate other findings that suggest community-level power dynamics and a lack of “family-centric community engagement strategies” serve as barriers to community engagement^36^ and participation.^20^

Incentives, or rather the lack of incentives, are often cited in the literature as a barrier to community engagement. Limited incentives have been noted as barriers to community engagement in vaccination campaigns generally^36^ and polio eradication programs specifically.^34^ One study examining barriers and facilitators of community engagement in the context of polio eradication programs even observed that a major challenge of community engagement was waning motivation due to a lack of financial incentives.^37^ The lack of small incentives and renumeration has also been noted as a major challenge to community engagement and participation of volunteer community health councils in the context of malaria elimination programming.^38^ Incentives have also been documented as a facilitator of community engagement. Financial incentives were one intervention used late in the smallpox eradication campaign that helped sustain interest among affected communities and health workers, kept health workers vigilant, and combated fatigue and complacency as cases waned.^6^

The provision of small financial incentives is a complex issue that deserves careful country- and community-specific deliberation and consultation, and there may be other options for motivating community engagement. For example, the African Program for Onchocerciasis Control identified community-directed distributors (CDDs) as being instrumental in providing health education and distributing ivermectin, but CDDs demanded renumeration for treating more than one section of the community.^39^ To overcome these demands, it was decided that every self-identified kinship or neighborhood group would select its own CDDs to work within its respective kinship zones. Under this strategy, CDD performance met expectations, and dropout rates remained low; the majority of those who did drop out were not actually selected by their community members.^39^

Several of the preferences and recommendations for community engagement set forth by our participants are corroborated by other studies as well. The selection of community volunteers through an open and fair selection process was also noted as an important factor of community buy-in for community engagement in polio eradication programming in Ethiopia,^40^ and engaging religious leaders and authority (e.g., referencing passages from religious text) has been noted as a strategy for developing meaningful community mobilization and engagement in polio eradication programming in Ghana.^41^ The need to address context-specific barriers affecting community engagement and to involve and empower community members in the planning and implementation of program activities within the context of eradication and health programs is also a salient theme elsewhere in the literature.^7^^,^^34^^,^^42^

### Implications of findings for last mile disease eradication programs: Data-derived recommendations.

Our findings pinpoint some key behavioral factors influencing community engagement that provide data-driven insights that the PNEVG-T and polio-related initiatives can use to inform refinements to their community engagement approaches and that other global stakeholders may also leverage to further develop their last mile disease eradication learning agendas. Below, we present a list of recommendations for community engagement within the context of disease eradication programs that we derived from the barriers and facilitators indicated by the participants of this operational research. Specific recommendations for community engagement in the context of the PNEVG-T and polio eradication initiatives, which may also be relevant to other last mile disease eradication programs, include the following:

#### Ensure engagement with the endogenously established structures the community values as well as the exogenously established structures created by the program.

Endogenously established community groups and structures may have more influence and reach within communities than exogenously established groups and structures.^43^ Program administrators can increase ownership, governance, and buy-in of engagement initiatives and community-based program interventions among community members by further leveraging and engaging with existing community structures that the communities themselves have established organically. Engagement with endogenously established structures may be particularly important if and when financial or material incentives are used to promote the community’s engagement with disease eradication programs and their adoption of disease-preventive program interventions.

#### Improve community engagement approaches and resulting community buy-in, ownership, and governance thereof by involving communities in the co-design, implementation, and monitoring of refined interventions.

The following strategies are recommended:

1. Engage communities in all phases of the program cycle, as community co-design of interventions has been observed to improve community buy-in and uptake.^44^^,^^45^ Involve and collaborate with community members in all aspects of the program cycle—from conception and design of refined community engagement interventions to targeting and implementation, troubleshooting and decision-making, and monitoring of progress toward program goals. Lessons can be learned from prior endeavors in low- and middle-income settings where intervention communities were engaged in the reflection of baseline health results, design of intervention activities and strategies, and trainings of community volunteers as intervention organizers and health educators.^45^ Best practices for participatory action learning and action research for community engagement^46^ may also help guide successful interactions with communities at various points along the program cycle.
2. Move beyond information-based interventions aimed at increasing awareness to address other factors that have more influence over behavior.^47^ Last mile disease eradication programs should modify the design, targeting, and implementation of their community engagement approaches and related program interventions so they address more proximal behavioral factors (i.e., those more likely to actually influence community participation and the adoption of disease-preventive behaviors), not just those related to awareness of vaccination activities and cash rewards for reporting of GW and tethering of animals. Evidence-based guidance exists about which specific intervention techniques program implementers can and should use to address certain behavioral factors effectively.^15^^,^^48^ Related interventions can enhance information-based interventions to incorporate other intervention techniques that address the factors influencing behavior, which can more readily facilitate behavior change (e.g., addressing misconceptions and false factual beliefs about perceived vaccine-related side effects, leveraging empirical and normative expectations, shifting community attitudes, improving trust and respect for community volunteers, and enhancing women’s agency and collective agency more broadly).
3. Refine community engagement approaches so they are gender sensitive and responsive and proactively address gender dynamics. Factors influencing women’s participation and engagement with health and development programs were observed to align with those influencing the uptake of disease-preventive behaviors among women. By addressing gender issues and factors influencing women’s engagement, disease eradication programs will likely observe progress toward their core program goals.

#### Expand community engagement infrastructure to more effectively reach highly mobile and transient populations.

Last mile disease eradication programs should consider establishing mechanisms to actively engage highly mobile, transient, and nomadic communities. For example, the South Sudan GWEP embeds GW Surveillance Officers within mobile cattle-rearing groups, many of whom are members of the mobile groups. The recruitment of community volunteers for vaccination and intensive surveillance for GW along with public criers in nomadic communities could help expand the community engagement infrastructure among these populations and facilitate the reach of the programs. The development of dynamic population movement maps (i.e., maps created with community members [and, in some instances, with the subsequent use of mapping software] that depict how and why people move over space and time and the routes they typically use to move from one location to another) created with the chiefs and elders of these nomadic communities may help the healthcare system have a better understanding of when and where highly mobile populations may be moving into and out of certain areas for improved planning and implementation of program activities to ensure coverage among these populations.

#### Support health systems strengthening (e.g., Chad’s Community Health Strategy) as a means of improving the infrastructure in place for engaging communities.

The PNEVG-T and the EPI may help support the Chadian Ministry of Health’s Community Health Strategy (i.e., the recruitment of a community relais in every village) to further expand the trained network of community-based personnel who can support the EPI’s vaccination efforts—polio and otherwise—and GW surveillance and response. Aside from their role in primary healthcare, these community relais could also be leveraged to reinforce vaccination activities in remote villages lacking access to vaccination services and reinforce acute flaccid paralysis surveillance activities. If properly trained, these community relais could also be a way for community members living in remote areas to be in contact with essential health services and health-related information. In addition to continuing to support more vertical program implementation efforts, disease eradication programs may also contribute to cross-program collaborations that support community engagement from a systems perspective to address community health needs more broadly.

#### Consider and design incentives carefully.

Incentives, particularly financial incentives, should be designed with thoughtfulness and care and draw on theory and evidence, including but not limited to findings from formative research with target communities and knowledge of norms.^49^ Behavioral attributes and community characteristics need to be taken into account^50^ when determining whether and when financial incentives may be appropriate interventions to implement. These considerations are important for the design of program interventions and incentives, generally speaking, but are particularly important to acknowledge given that incentives were identified as both barriers to and facilitators of community engagement behavior.

### Strengths and limitations.

There were several strengths and limitations of this operational research. With regard to strengths, our employment of participatory qualitative data collection methods yielded rich data that reflected participant perspectives and experiences and permitted examinations of nuanced complexities between behavioral factors. Our use of qualitative methods that were grounded in relevant behavioral and decision theories helped reveal factors that influence community engagement that may have been overlooked by quantitative methods.

With regard to limitations, this operational research was executed only in a single district in Chad, a country that is diverse ethnically, even among African countries. Although the study team engaged numerous subgroups from different villages within the district of Bongor to obtain information reflecting an array of perspectives, the data generated from this one district may not align with the infrastructure, events, and lived experiences observed by community members in other districts of Chad. The transferability of these results remains unknown, and our results should be interpreted accordingly. Participant recruitment was particularly challenging in some study sites (i.e., Dor), which had implications for the number and composition of individuals who participated in some data collection activities in those communities. To guarantee anonymity and confidentiality, the FGD and IDI questions were framed in a manner that allowed participants to talk about their experiences as well as the experiences of others. In some instances, this approach may have limited our ability to attribute data to a specific subgroup and, consequently, may have biased our subgroup and intersectionality analyses. Finally, FGD and IDI facilitators did not speak the local dialect (Massa/Arabic) and therefore relied on notetakers to translate the facilitator’s questions from French to Massa/Arabic and the participant’s responses from Massa/Arabic to French for the benefit of the facilitator. As a result, some concepts may have gotten lost in translation during the facilitation of the FGDs and some IDIs, and the notetakers may not have captured all pertinent points mentioned by the participants in their field notes. That said, transcriptions of audio recordings from the FGDs and IDIs served as the primary data source for the thematic analyses.

## CONCLUSION

In conclusion, the key findings presented herein can help stakeholders better understand the nuanced factors influencing community engagement behavior in the Bongor District of Chad. Stakeholders at various levels may leverage these data-driven insights to inform the development of evidence-based program and policy decisions to further refine and enhance community engagement for better intervention design and implementation, bi-directional communication with program participants, improved uptake of program interventions, and more effective interruption of disease transmission. Although the findings presented reflect program- and context-specific findings and recommendations, they may also help inform and refine learning agendas for other last mile disease eradication programs. As disease eradication initiatives make progress toward their goals, the engagement of communities in the detection, prevention, and response to the final cases of these diseases will remain integral to the success of these efforts.

## Supplemental Materials

10.4269/ajtmh.23-0635 Supplemental Materials

## References

[1] Morgan MA Lifshay J, 2006. *Community Engagement in Public Health*. Available at: https://www.schoolhealthcenters.org/wp-content/uploads/2011/09/community_engagement.pdf. Accessed July 1, 2023.

[2] Brunton G Thomas J O’Mara-Eves A Jamal F Oliver S Kavanagh J, 2017. Narratives of community engagement: A systematic review-derived conceptual framework for public health interventions. BMC Public Health 17: 944. 29228932 10.1186/s12889-017-4958-4 PMC5725895

[3] World Health Organization , 1978. Declaration of Alma-Ata. Alma-Ata, USSR. Geneva, Switzerland: World Health Organization. Available at: https://www.who.int/publications/i/item/9241800011. Accessed July 7, 2023.

[4] Henderson DA, 2011. The eradication of smallpox–An overview of the past, present, and future. Vaccine 29 *(Suppl 4):* D7–D9. 22188929 10.1016/j.vaccine.2011.06.080

[5] Richards FO Ruiz-Tiben E Hopkins DR, 2011. Dracunculiasis eradication and the legacy of the smallpox campaign: What’s new and innovative? What’s old and principled? Vaccine 29 *(Suppl 4):* D86–D90. 22185836 10.1016/j.vaccine.2011.07.115

[6] Tarantola D Foster SO, 2011. From smallpox eradication to contemporary global health initiatives: Enhancing human capacity towards a global public health goal. Vaccine 29 *(Suppl 4):* D135–D140. 22185838 10.1016/j.vaccine.2011.07.027

[7] Andrus JK Perry HB, 2019. Community engagement, ownership, and civil society organizations in polio eradication. Am J Trop Med Hyg 101 *(Suppl):* 1–3. 10.4269/ajtmh.19-0529 PMC6776102 31760981

[8] Akinyemi OO Adebayo A Bassey C Nwaiwu C Kalbarczyk A Fatiregun AA Alonge OO Owoaje E, 2021. Assessing community engagement in Nigeria polio eradication initiative: Application of the Consolidated Framework for Implementation Research. BMJ Open 11: e048694. 10.1136/bmjopen-2021-048694 PMC8354285 34373306

[9] Ghinai I Willott C Dadari I Larson HJ, 2013. Listening to the rumours: What the northern Nigeria polio vaccine boycott can tell us ten years on. Glob Public Health 8: 1138–1150. 24294986 10.1080/17441692.2013.859720 PMC4098042

[10] Hopkins D Choffnes ER Relman DA The Causes and Impacts of Neglected Tropical and Zoonotic Diseases: Opportunities for Integrated Intervention Strategies. Washington, DC: The National Academies Press, 604. 21977543

[11] World Health Organization , 2020. Global Polio Eradication Initiative Applauds WHO African Region for Wild Polio-Free Certification [press release]. Geneva, Switzerland. Available at: https://www.who.int/news/item/25-08-2020-global-polio-eradication-initiative-applauds-who-african-region-for-wild-polio-free-certification. Accessed January 28, 2022.

[12] Bigouette J Wilkinson A Tallis G Burns C Wassilak S Vertefeuille J, 2021. Progress toward polio eradication—Worldwide, January 2019–June 2021. MMWR Morb Mortal Wkly Rep 70: 1129–1135. 34437527 10.15585/mmwr.mm7034a1 PMC8389387

[13] Barber C Mueller CT Ogata S, 2013. Volunteerism as purpose: Examining the long-term predictors of continued community engagement. Educ Psychol 33: 314–333.

[14] World Health Organization , 2022. *Polio (Pol3) Immunization Coverage Estimates by Country*. Available at: https://apps.who.int/gho/data/node.main.A831. Accessed March 18, 2024.

[15] Michie S van Stralen MM West R, 2011. The behaviour change wheel: A new method for characterising and designing behaviour change interventions. Implement Sci 6: 42. 21513547 10.1186/1748-5908-6-42 PMC3096582

[16] Collins CS Stockton CM, 2018. The central role of theory in qualitative research. Int J Qual Methods 17, doi: 10.1177/1609406918797475.

[17] Zanbar L Ellison N, 2019. Personal and community factors as predictors of different types of community engagement. J Community Psychol 47: 1645–1665. 31269249 10.1002/jcop.22219

[18] Ferrer R Klein WM, 2015. Risk perceptions and health behavior. Curr Opin Psychol 5: 85–89. 26258160 10.1016/j.copsyc.2015.03.012 PMC4525709

[19] Harclerode MA Lal P Vedwan N Wolde B Miller ME, 2016. Evaluation of the role of risk perception in stakeholder engagement to prevent lead exposure in an urban setting. J Environ Manage 184: 132–142. 27477350 10.1016/j.jenvman.2016.07.045

[20] Geyer RE , 2020. Gender norms and mass deworming program access in Comé, Benin: A qualitative assessment of gender-associated opportunities and challenges to achieving high mass drug administration coverage. PLoS Negl Trop Dis 14: e0008153. 32302298 10.1371/journal.pntd.0008153 PMC7164589

[21] Kabeer N, 1999. Resources, agency, achievements: Reflections on the measurement of women’s empowerment. Dev Change 30: 435–464.

[22] Van Eerdewijk A Wong F Vaast C Newton J, Tyszler M, Pennington A; Bill & Melinda Gates Foundation , 2017. White Paper: A Conceptual Model of Women and Girls’ Empowerment. Amsterdam, The Netherlands: Royal Tropical Institute.

[23] Eissler S Heckert J Myers E Seymour G Sinharoy S Yount K, 2022. Measuring women’s empowerment: Gender and time-use agency in Benin, Malawi and Nigeria. Dev Change 53: 1010–1034.

[24] Kirchherr J Charles K, 2018. Enhancing the sample diversity of snowball samples: Recommendations from a research project on anti-dam movements in Southeast Asia. PLoS One 13: e0201710. 30133457 10.1371/journal.pone.0201710 PMC6104950

[25] Quinlan MB Liamputtong P Handbook of Research Methods in Health Social Sciences. Singapore: Springer Singapore, 1431–1446.

[26] Braun V Clarke V, 2006. Using thematic analysis in psychology. Qual Res Psychol 3: 77–101.

[27] MacQueen KM McLellan E Kay K Milstein B, 1998. Codebook development for team-based qualitative analysis. CAM Journal. 10: 31–36.

[28] Nowell LS Norris JM White DE Moules NJ, 2017. Thematic analysis: Striving to meet the trustworthiness criteria. Int J Qual Methods 16, doi: 10.1177/1609406917733847.

[29] Thomas J Harden A, 2008. Methods for the thematic synthesis of qualitative research in systematic reviews. BMC Med Res Methodol 8: 45. 18616818 10.1186/1471-2288-8-45 PMC2478656

[30] Jordan A, Kunkel D, Manganello J, Fishbein M, eds. 2008. *Reliability for content analysis in Media Messages and Public Health: A Decisions Approach to Content Analysis (1st ed.).* New York: Routledge. Available at: 10.4324/9780203887349. Accessed June 17, 2024.

[31] Helma L, 2015. Intersectionality as method. DiGeSt Journal of Diversity and Gender Studies. 2: 39–44.

[32] Miaskiewicz T Kozar KA, 2011. Personas and user-centered design: How can personas benefit product design processes? Des Stud 32: 417–430.

[33] Baltzell K Harvard K Hanley M Gosling R Chen I, 2019. What is community engagement and how can it drive malaria elimination? Case studies and stakeholder interviews. Malar J 18: 245. 31315631 10.1186/s12936-019-2878-8 PMC6637529

[34] Agrawal P Neel A Deresse AS Gerber S Alonge O, 2023. Facilitators and barriers to community engagement in the Global Polio Eradication Initiative: A mixed methods study. PLOS Glob Public Health. 3: e0001643. 37027352 10.1371/journal.pgph.0001643 PMC10081736

[35] Losey L , 2019. The CORE Group Polio Project: An overview of its history and its contributions to the Global Polio Eradication Initiative. Am J Trop Med Hyg 101 *(Suppl):* 4–14. 31760971 10.4269/ajtmh.18-0916 PMC6776098

[36] Dutta T Agley J Meyerson BE Barnes PA Sherwood-Laughlin C Nicholson-Crotty J, 2021. Perceived enablers and barriers of community engagement for vaccination in India: Using socioecological analysis. PLoS One 16: e0253318. 34170920 10.1371/journal.pone.0253318 PMC8232440

[37] Chimpololo A Burrowes V, 2019. Use of social mobilization and community mobilizers by non-governmental health organizations in Malawi to support the eradication of polio, improve routine immunization coverage, and control measles and neonatal tetanus. Am J Trop Med Hyg 101 *(Suppl):* 85–90. 31760969 10.4269/ajtmh.19-0021 PMC6776103

[38] Bardosh K , 2023. Evaluating a community engagement model for malaria elimination in Haiti: Lessons from the community health council project (2019–2021). Malar J 22: 47. 36759860 10.1186/s12936-023-04471-z PMC9910254

[39] Katabarwa MN Habomugisha P Richards FO Jr Hopkins D, 2005. Community-directed interventions strategy enhances efficient and effective integration of health care delivery and development activities in rural disadvantaged communities of Uganda. Trop Med Int Health 10: 312–321. 15807794 10.1111/j.1365-3156.2005.01396.x

[40] Stamidis KV Bologna L Bisrat F Tadesse T Tessema F Kang E, 2019. Trust, communication, and community networks: How the CORE Group Polio Project community volunteers led the fight against polio in Ethiopia’s most at-risk areas. Am J Trop Med Hyg 101 *(Suppl):* 59–67. 10.4269/ajtmh.19-0038 PMC6776093 31760978

[41] Lohiniva A-L Nurzhynska A Alhassan H Shetye M Ayiku P, 2022. Understanding factors influencing polio vaccine uptake in Ghana—Developing meaningful community mobilization and engagement strategies in collaboration with religious leaders. Am J Trop Med Hyg 107: 1345–1350. 36315999 10.4269/ajtmh.22-0271 PMC9768250

[42] Silumbwe A Halwindi H Zulu JM, 2019. How community engagement strategies shape participation in mass drug administration programmes for lymphatic filariasis: The case of Luangwa District, Zambia. PLoS Negl Trop Dis 13: e0007861. 31774820 10.1371/journal.pntd.0007861 PMC6905562

[43] Holcombe SH, 2014. Donors and exogenous versus endogenous development. Dev Pract 24: 750–763.

[44] Bonham-Werling J Passmore S Hendricks K Bednarz L Faust V Talaga A Maureen S, 2021. Co-designing to advance community health and health equity in Wisconsin: Building the Neighborhood Health Partnerships Program. J Clin Transl Sci 5: e87.

[45] Andersson N , 2015. Evidence based community mobilization for dengue prevention in Nicaragua and Mexico (Camino Verde, the Green Way): Cluster randomized controlled trial. BMJ 351: h3267. 26156323 10.1136/bmj.h3267 PMC4495677

[46] Wood L Zuber-Skerritt O Wood L Action Learning and Action Research: Genres and Approaches. Leeds, England: Emerald Publishing Limited, 193–206.

[47] Flay BR Snyder F Petraitis J Crosby R Kegler MC DiClemente RJ Emerging Theories In Health Promotion Practice And Research. New York: Jossey-Bass, 451–510.

[48] Abraham C Michie S, 2008. A taxonomy of behavior change techniques used in interventions. Health Psychol 27: 379–387. 18624603 10.1037/0278-6133.27.3.379

[49] Lapinski MK Kerr JM Zhao J Shupp RS, 2017. Social norms, behavioral payment programs, and cooperative behaviors: Toward a theory of financial incentives in normative systems. Hum Commun Res 43: 148–171.

[50] Lapinski MK Rimal RN DeVries R Lee EL, 2007. The role of group orientation and descriptive norms on water conservation attitudes and behaviors. Health Commun 22: 133–142. 17668993 10.1080/10410230701454049

