# Supplementary material for: Factors Influencing Community Engagement during Guinea Worm and Polio Eradication Endgames in Chad: Recommendations for “Last Mile” Programming
Source: Am J Trop Med Hyg. 2024 Jul 9;111(3 Suppl):36–48. doi: 10.4269/ajtmh.23-0635 (PMC11376110; doi:10.4269/ajtmh.23-0635)
Supplement: Supplemental Materials [file tpmd230635.SD1.pdf]

SUPPLEMENTAL MATERIALS

Factors influencing community engagement during Guinea worm and polio eradication endgames in Chad: Recommendations for 'last mile' programming

Maryann G. Delea, Lalique Browne, Severin Kaji, Adam Weiss, Tchindebet Ouakou

Supplemental Methods

Discussion and interview guide development and refinement

The study team designed discussion guides for use during FGDs and interview guides for use during IDIs, each of which contained prompts, probes, and follow-up questions that the team developed *a priori* to collect data on the topics and constructs outlined above. After the initial development of the FGD and IDI guides, the study team tested the guides during two waves of piloting conducted in two separate non-study villages. Results from the piloting phase were not included in the analytical sample. The study team refined the guides after each wave of piloting to generate the initial field-ready guides. Throughout the official data collection phase, the team revised the guides iteratively based on insights discussed during debriefings (see 'Debriefings'). In addition to the prompts, probes, and following-up questions in the guides, discussion group facilitators and interviewers asked other relevant questions and probes that followed the organic flow of the conversation and participants' responses.

Debriefings

After each data collection activity, the teams convened activity-specific debriefing sessions. The entire study team convened full debriefings on the day that followed each day of data collection as well. These full debriefing discussions were convened to identify emerging themes, determine whether saturation had been met, decide whether prompts needed to be dropped (e.g., if saturation had been met), and discuss whether new questions should be added to delve deeper into emerging themes.

Transcription and translation of audio files

Five transcribers who were fluent in Massa, Arabic, and French transcribed all audio files to generate IDI and FGD transcripts. For quality assurance purposes, transcribers were required to listen to and independently transcribe a five-minute segment of an audio file, selected by the study team, that was transcribed by another transcriber. The study team reviewed the two transcriptions for quality and comparability, and provided feedback for improvement and re-transcription, as needed. This quality assurance exercise was completed for every audio file/transcript.

Table S1. Participant characteristics

Basic characteristics – All participants	n (%)
**Central level N=6**	
Age, in years - median (IQR)	57.5 (55 - 64)
Men	5 (83)
**District level N=5**	
Age, in years - median (IQR)	45 (44 - 48)
Men	5 (100)
**Local level N=6**	
Age, in years - median (IQR)	47 (35 - 51)
Men	6 (100)
**Community level N = 110 (FGD n = 98, IDI n = 12)**	
Age, in years - median (IQR)	37 (30 - 49)
Men	62 (56)
**Detailed characteristics and demographics — Community level participants, N = 110**	
**Religion**	
Christian	98 (89)
Muslim	4 (4)
Animist	7 (6)
**Ethnicity**	
Massa	67 (61)
Marba	21 (19)
Gabri	14 (13)
Other (Arabe, Moussey, Kiam, Ham, Nantchere)	8 (7)
**Occupation**	
Farmer	88 (80)
Housewife	7 (6)
Sales/Business	4 (4)
Other(Animal herder, student) fisher)	11 (10)
**Member of a community association and/or a volunteer**	
No	53 (48)
Yes	54 (49)
Refused to answer	3 (2)
**Involvement in community group**	
Farmers' group member	36 (66)
Community Volunteer for Intensive Surveillance of Guinea worm	7 (13)
Community development association member	5 (9)
Traders group member	1 (2)
Other	5 (9)
**Educational attainment**	
No schooling	42 (38)
Primary	26 (24)
Secondary	35 (32)
Higher	7 (6)
**Marital status**	
Married	92 (84)
Widowed	11 (10)
Single	6 (5)
Divorced	0 (0)
**Household characteristics**	
Average number of people living in the household (median, IQR)	8 (6 – 12)
Average time, in minutes, to reach the primary drinking water source, fetch & come back (median, IQR)	5 (3 – 10)
Average time, in minutes, to reach the health center (median, IQR)	40 (15 – 180)
**Primary drinking water source**	
Tubewell / borehole	99 (90)
Unprotected dug well	6 (5)

Surface water	1 (1)
Machine dug deep well	2 (2)
Protected dug well	1 (1)
**Type of treatment sought in sickness**	
Purchase medicines from a street vendor	62 (56)
Consult a health professional at a health center/hospital	26 (24)
Consult a community health agent	8 (7)
Prepare traditional medicines	8 (7)
Consult a traditional healer	5 (4)
